# Comparing NISAR (Using Sentinel-1), USDA/NASS CDL, and Ground Truth Crop/Non-Crop Areas in an Urban Agricultural Region

**DOI:** 10.3390/s23208595

**Published:** 2023-10-20

**Authors:** Simon Kraatz, Brian T. Lamb, W. Dean Hively, Jyoti S. Jennewein, Feng Gao, Michael H. Cosh, Paul Siqueira

**Affiliations:** 1 USDA ARS Hydrology and Remote Sensing Laboratory, Beltsville, MD 20705, USA; feng.gao@usda.gov (F.G.); michael.cosh@usda.gov (M.H.C.); 2 USGS Lower Mississippi-Gulf Water Science Center, Coram, NY 11727, USA; blamb@usgs.gov; 3 USGS Lower Mississippi-Gulf Water Science Center, Beltsville, MD 20705, USA; whively@usgs.gov; 4 USDA ARS Sustainable Agricultural Systems Laboratory, Beltsville, MD 20705, USA; jyoti.jennewein2@usda.gov; 5 Department of Electrical and Computer Engineering, University of Massachusetts Amherst, Amherst, MA 01003, USA; siqueira@umass.edu

**Keywords:** Sentinel-1, land use and coverage, agriculture, radar

## Abstract

A general limitation in assessing the accuracy of land cover mapping is the availability of ground truth data. At sites where ground truth is not available, potentially inaccurate proxy datasets are used for sub-field-scale resolution investigations at large spatial scales, i.e., in the Contiguous United States. The USDA/NASS Cropland Data Layer (CDL) is a popular agricultural land cover dataset due to its high accuracy (>80%), resolution (30 m), and inclusions of many land cover and crop types. However, because the CDL is derived from satellite imagery and has resulting uncertainties, comparisons to available in situ data are necessary for verifying classification performance. This study compares the cropland mapping accuracies (crop/non-crop) of an optical approach (CDL) and the radar-based crop area (CA) approach used for the upcoming NASA-ISRO Synthetic Aperture Radar (NISAR) L- and S-band mission but using Sentinel-1 C-band data. CDL and CA performance are compared to ground truth data that includes 54 agricultural production and research fields located at USDA’s Beltsville Agricultural Research Center (BARC) in Maryland, USA. We also evaluate non-crop mapping accuracy using twenty-six built-up and thirteen forest sites at BARC. The results show that the CDL and CA have a good pixel-wise agreement with one another (87%). However, the CA is notably more accurate compared to ground truth data than the CDL. The 2017–2021 mean accuracies for the CDL and CA, respectively, are 77% and 96% for crop, 100% and 94% for built-up, and 100% and 100% for forest, yielding an overall accuracy of 86% for the CDL and 96% for CA. This difference mainly stems from the CDL under-detecting crop cover at BARC, especially in 2017 and 2018. We also note that annual accuracy levels varied less for the CA (91–98%) than for the CDL (79–93%). This study demonstrates that a computationally inexpensive radar-based cropland mapping approach can also give accurate results over complex landscapes with accuracies similar to or better than optical approaches.

## 1. Introduction

Agricultural land use has important implications for food security and Earth system processes, particularly the nitrogen (e.g., fertilizer), carbon (e.g., biomass), and water (e.g., evapotranspiration) cycles [1,2,3,4]. Many practical agricultural monitoring applications need frequent (<weekly) large-scale (global) observations at moderate (<30 m) to high (<4 m) spatial resolutions [5]. The most commonly used observations to meet these demands are from optical satellite sensors such as Landsat, Sentinel-2, and others [5]. Optical sensors have many benefits, such as being supplied in an analysis-ready data (ARD) format, which, notably, also includes data quality flags at each pixel. Data quality flags hold important information on clouds, cloud shadows, or other transient surface conditions such as snow. The main drawback of optical instruments is that they cannot view the Earth’s surface when cloudy. Cloud cover is highly variable in time and space, and important agricultural events such as green-up and harvest dates may not be accurately detected [6,7]. Furthermore, spaceborne optical data usually only captures two-dimensional information, inferring properties such as biomass by indirect means, such as canopy closure [8]. Most optical-based crop mapping products use decision tree and random forest approaches [9,10,11,12,13]. These approaches are computationally expensive, given that they first require model training and then must step through the decision tree at each pixel. Although current human and computational requirements are sufficient for making annual cropland maps, it is likely that associated costs make it challenging to produce more frequent maps, which could reduce the latency by many months. Furthermore, these approaches mainly focus on the peak growing season, and classifications can be difficult if this period coincides with cloudy conditions [14].

Radar is an active sensing approach where a power source on the satellite provides the energy for emitting and receiving the signal, and radar systems collect data of equal quality irrespective of the time of day. Radar also uses microwave frequencies (~1–30 GHz), corresponding to centimeter-scale wavelengths (e.g., 1–30 cm). The microwave frequency range is highly sensitive to water due to its absorption/emission bands falling in this range and thus presents an excellent tool for monitoring water dynamics such as soil moisture [15]. Its wavelength also makes radar less sensitive to atmospheric effects, allowing reliable observations of the surface even in cloudy conditions [16,17]. Commonly used spaceborne radar instruments measure the returned (backscattered) portion of the electromagnetic waves after they have interacted with the landscape. The radar then detects a voltage wave represented as a phasor (complex number), having an amplitude and phase. 

In the most commonly used configuration, radar data is sent and received in horizontal (H) or vertical (V) polarization, yielding four sent/received combinations: HH, HV, VH, and VV. Backscatter amplitude, phase, and polarization are sensitive to the particular landscape element’s properties, allowing for landcover classifications. However, radar classifications are more limited than optical classifications due to spaceborne radar normally only having a single frequency, as compared to optical instruments that have a dozen or more bands [11,18]. 

For polarimetry work, backscatter data is first converted to power units normalized by area, referred to as radar cross section (RCS), ahead of use. Because the scattering is a three-dimensional process and may penetrate plants and soils ahead of being returned, radar data allow users to infer three-dimensional information relating to soil and plant properties such as vegetation structure, soil and vegetation water content, and Earth surface deformations [19,20,21,22,23,24,25]. Although radar sensing can be a powerful tool, its most substantial drawback is that radar imagery is usually not provisioned as ARD [26]. Data often need extensive preprocessing and quality control by the end users. For example, radar data do not have data quality flags at each pixel and may potentially incorporate noise unrelated to Earth surface processes, such as radio frequency interference (RFI). This can be a hindrance for large-scale cloud computing efforts as erroneous pixels cannot be readily screened out. Radar data also often require additional processing for data to be properly geolocated and calibrated, necessitating user knowledge of tools such as the European Space Agency’s (ESA) Sentinel Application Platform (SNAP) software [27]. For some types of analyses (polarimetry and interferometry), further processing is required using tools such as PolSARPro, GAMMA software, or NASA’s Interferometric Synthetic Aperture Radar (SAR) Scientific Computing Environment (ISCE) [28,29,30]. However, data providers are taking on these processing requirements, making analyses less computationally expensive to users. For example, Google Earth Engine freely hosts Sentinel-1 data that were processed by SNAP [31]. Users may also request free Sentinel-1 on-demand cloud processing for terrain flattening or interferometric coherence and phase calculation using Alaska Satellite Facility’s (ASF) Vertex platform [32,33]. On-demand processing is performed using GAMMA but has a monthly quota. A key objective of future radar missions such as the NISAR is to make ARD readily available (including terrain flattened and interferometric products), allowing users comparable data processing options to those available for optical imagery but without data quality flags [19].

This work focuses on comparing crop/non-crop mapping performance of the optical-based USDA/NASS Cropland Data Layer (CDL) and the radar-based NISAR crop area science algorithm (CA) at the C-band against ground truth data. There are other potential land cover datasets that could be used, but the CDL is overall the most useful for this radar-based study, owing to its combination of appropriate resolution (30 m), latency (annual) and stratification of crops (>50 crop types). Comparatively, the National Land Cover Database provides the same gridding (30 m) but is only updated every five years and has no stratification of crops [34]. Other products with the same latency as the CDL are the global land cover products from NASA’s Moderate Resolution Imaging Spectroradiometer (MODIS, MCD12Q1.061, 500 m pixels) or ESA’s Sentinel-2 based CGLS-LC100 product (100 m pixels), but they are coarser than the CDL and do not have crop stratification. There are also finer-gridded global products, such as ESA’s WorldCover 10 m 2020 v100 product, but they are not updated every year and do not have crop stratification [35,36]. The detailed breakdown of agricultural crop types can be useful for evaluating the strengths and weaknesses of NISAR’s CA product [37,38].

While there have been multiple studies of NISAR’s CA in the past covering both the L- and C- band, all but one was limited to using a proxy for ground truth data [37,38,39,40,41,42,43]. The only study comparing NISAR’s CA approach to ground truth was conducted in Germany and did not include comparisons to any of the large-scale optical map products [41]. Comparisons of the CA product with optical products were usually found to have over 80% agreement, which is also the accuracy requirement for NISAR CA [37,38,39,40,42,43]. The CA approach has also been tested by land cover class, showing that it generally performs well over most crops and forests but has some difficulty in detecting grassland, pasture, and urban land cover as non-crop [38,43]. Most previous studies only stratified evaluations by crop and non-crop. However, the two studies were also stratified by different crop types. One study was for an agricultural region in Canada [38], and the other was for 100 1-by-1-degree tiles in the contiguous USA [43]. In both cases, only the most prevalent row crops were considered for each region, such as corn, soybeans, wheat, barley, oats, and canola. Both studies showed that the median and interquartile range for the coefficient of variation (CV) values had considerable overlap for crops. Therefore, both studies concluded that the approach used is not suitable for distinguishing between different crops. However, there were substantial differences between those metrics from the crop versus non-crop classes, and the studies agreed that the CV approach is suitable for obtaining relatively accurate (i.e., often >80%) crop/non-crop classifications.

The CDL has been exhaustively tested and was shown to have good agreement (>85%) with ground truth, but the reported accuracies vary by region and crop type, and the intermixing of scattered crop pixels in non-crop areas remains problematic [44]. The CDL uses the USDA Farm Service Agency’s (FSA) common land unit (CLU) data as ground truth. CLUs are polygons identifying contiguous cropland management areas. In the process of generating the CDL product, the CLU polygons are rasterized, and those pixels are then selected from the training and accuracy assessments [9]. The non-crop data in the CDL is taken from other data sources, such as the National Land Cover Database [34]. It is difficult to give a confident estimate on the CDL’s crop/non-crop mapping performance due to the stratified nature of the CDL’s data sources (NLCD + CLU) and how accuracies are provided (by crop type at CLU validation pixels).

In previous studies, accuracy evaluations of the NISAR CA approach were limited to optical cropland datasets that are not ground truth, such as the ACI and CDL. Therefore, it was only possible to assess the correspondence between radar and widely used optical cropland mapping approaches, but it was not possible to determine what the performance gap was between them. Unlike previous studies, we use ground truth data to evaluate the radar (CA) and optical (CDL) products, enabling us to report on the overall performance and relative strengths of each approach. It is also important to report on the results of optical versus radar-based approaches to contextualize the substantial cost and time reductions associated with radar-based cropland mapping, to better weigh the costs and benefit trade-offs between the two approaches and to decide which to employ. Reporting on radar-based cropland mapping performance is important and timely in light of impending (e.g., NISAR, BIOMASS) and future (e.g., ROSE-L) radar missions that may be used to further advance cropland mapping capabilities, given that there will be more frequent observations made and at multiple bands. The low-cost, all-weather capability of the CA approach is important because this allows it to produce multiple cropland maps each year (e.g., quarterly or better [19]), which greatly improves current agricultural monitoring capabilities.

## 2. Materials and Methods

### 2.1. Materials

Table 1 shows the datasets and tools used for conducting this study.

### 2.2. Study Area

**Figure 1:**
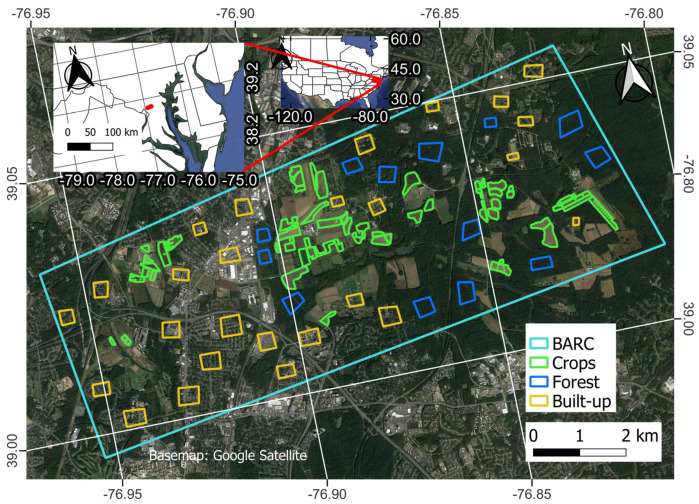
Study area of Beltsville Agricultural Research Center region in Maryland, USA. The region primarily consists of built-up, forest, and agricultural cropland use.

BARC fields are distributed throughout both the urban and relatively undeveloped parts of Beltsville, MD. This study focuses on a region encompassing most of both types of BARC fields, those that are encompassed within the polygon labeled “BARC” in Figure 1. It is situated in an area that has substantial coverage of agricultural fields (ranging from less than 1 to over 20 ha in size), built-up areas, and forests (Figure 1). BARC is a USDA Long-Term Agroecosystems Research (LTAR) site in Maryland, USA [45]. BARC was selected as the study site because it has a detailed long-term record of crop management information over a large number of fields representing many different crops, such as soybeans, corn and wheat. This is ideal for testing NISAR’s CA because performance may vary by crop type, and it also allowed us to verify which fields had been in active use each year (planted or harvested). The active use part is important, as a key premise of NISAR’s CA approach is that field management activities (e.g., tilling, growth, harvesting) during the growing season change RCS values more than over other land covers [41]. Another important aspect of BARC is that the region is large enough (~2670 ha) to encompass 50+ fields of ~0.8 ha or greater extent, making it suitable for the 30 m × 30 m remote sensing-based products evaluated in this paper (CDL and CA) [46]. Furthermore, the diverse representation of fields, forests and built-up land use makes BARC, and the surrounding area, an ideal location for assessing NISAR’s CA mapping limitations, given that NISAR’s CA was shown to be consistently accurate over fields and forests, but exhibits mixed results over built-up areas [38,43]. This location, therefore, provides an opportunity to evaluate the classification of urban areas, forest, and cropland.

It is important to note that the ‘crop’ polygons were digitized using GPS data and thus are not expected to contain any substantial amount of non-crop covers. Because the ‘forest’ polygons were hand-drawn over visually selected sites, they are also highly uniform but can include minor inhomogeneities due to roads and small clearings. The ‘built-up’ polygons consist of a combination of different land cover/landscape elements by design: they consist of built-up features like buildings (office, residential), paved surfaces (parking lots), and also intermixed lawns and trees that are common to residential and commercial areas.

### 2.3. Field Data

BARC maintains an extensive ground truth library of farm management practices in the FarmLogic system [47], which includes details on farm operations such as planting and harvesting dates, cultivars, and crop termination methods. This dataset also includes GIS data, such as shapefiles delineating field locations and extents, plus data on field classification (e.g., dairy, production, or research) and crop type. This database covers the entire period for which 12-day or more frequent Sentinel-1A/B observations were available over CONUS [48]—making it ideal for testing a Sentinel-1-based approach using time series of similar density as NISAR’s 12-day revisit. It is desirable to use dense time series to capture the effects of field management activities in a timely manner, such as harvesting and tilling [37,49].

The BARC field records in the FarmLogic needed additional data screening prior to analysis. It is important to note that the database also includes many small fields for research, resulting in a relatively high number of field dates in FarmLogic. FarmLogic contained about 400 to 500 field dates for harvesting operations each year and only covered 2019 to 2021. Planting data were available for the full range of this study from 2017 to 2021. However, planting entries had relatively greater variation in field dates, ranging from 400 to 800. This discrepancy stems from some fields not having any record or only one of planting or harvesting in some years, while other fields had multiple field operation entries within a single year. This can be explained by not every field management activity having been entered into the database. 

Working from this digital data record, we defined that an actively used field is one that had at least one planting or harvesting date in each year of the five-year period. This compromise was implemented to maximize the number of fields to be studied while also ensuring a reasonable level of confidence that each field was actively used for crops in each year studied. Furthermore, we required fields to be of reasonable size compared to the 30 m × 30 m pixels, imposing a 2-acre (0.81 ha) area requirement. Four highly instrumented research fields were also included due to their onsite long-term camera record indicating that these fields were in active use between 2017 and 2021, bringing the total to 54 [50]. Imposing a requirement of a field having at least one planting or harvesting record to be considered in this study results in some crop type omissions, as not all the crops or cover crops planted throughout the year may be represented in a full accounting based on the database only. Using the harvest and planting entries from 2019 as example, there were 19 soybean, 13 corn, 7 wheat, 5 rye, 4 cover crop, 2 grass, 1 mixed, 1 alfalfa, 1 brassica fields and 1 field labeled as miscellaneous.

**Figure 2:**
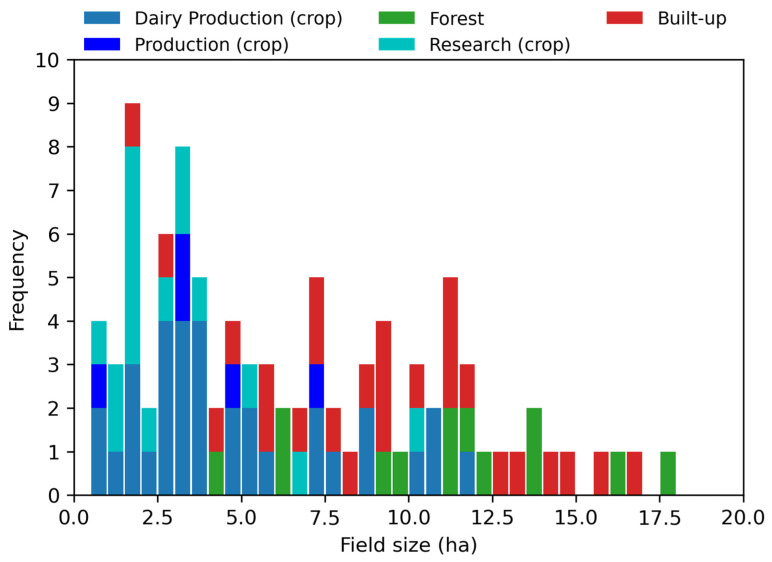
Field size distribution by field category in 0.5 ha bins.

For assessment purposes, it is also necessary to assess non-crop classification performance. Because this study focuses on classifying fields rather than pixels as crop or non-crop, regions of interest were hand-drawn over 13 forested and 26 built-up areas inside BARC (Figure 1). Figure 2 shows the size distribution of the crop and the non-crop fields, here broken down by dairy production (crops such as soybeans, corn, wheat, rye, and cover), production (crops, mainly soybeans), research (crops such as soybeans, corn, and rye), forest and built-up having mean areas of 4.8, 4.0, 3.1, 11.1 and 9.3 ha, respectively. In the context of U.S. agriculture, field sizes studied here are considerably smaller than the U.S. median value of 27.8 ha but closely resemble regional field sizes (median 6 ha) [51,52].

### 2.4. Remote Sensing Data

**Figure 3:**
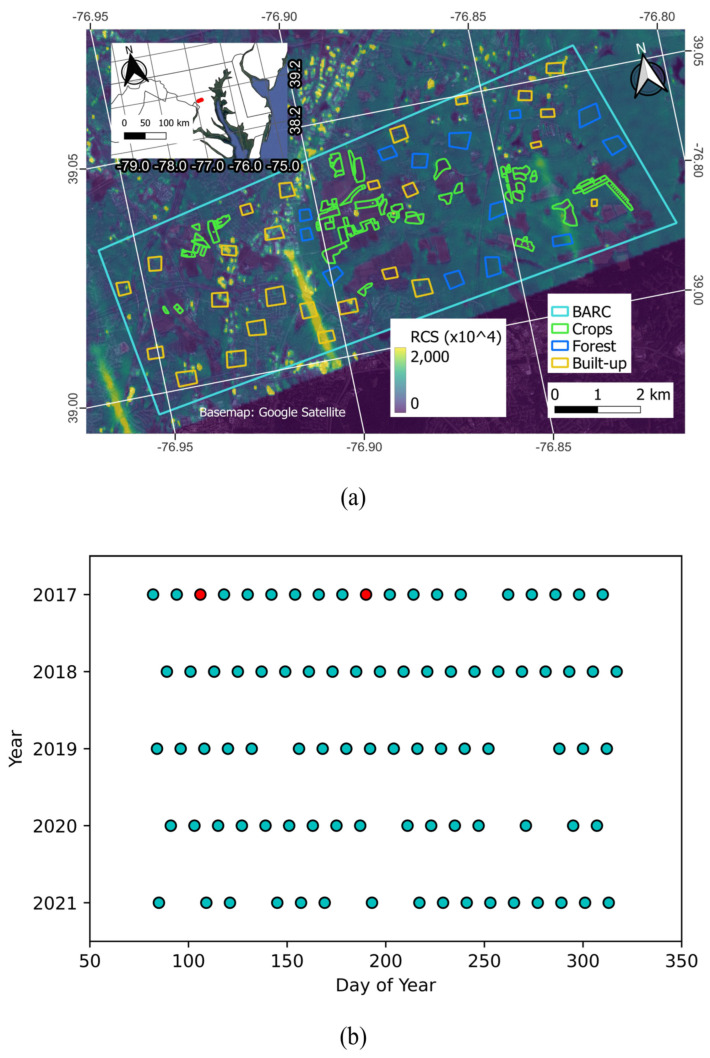
Example of impaired Sentinel-1 data quality over BARC on two of the dates (**a**), and inventory of all Sentinel-1 data used in the study ranging between 20 March and 16 November each year (blue-filled circles) (**b**). Red-filled circles in (**b**) indicate data impaired by RFI. The study site used data for ascending frame 125 at relative orbit 4 (ASF Vertex tiling). RCS stands for radar cross section in linear power units.

Cross-polarized (‘VH’) Sentinel-1 C-band data were used as input to the CA mapping approach. Data were processed using NASA’s ISCE software (ISCE-2, version 2.5.3) [29], using the rtcApp.py script but with a custom geocoding step. The rtcApp.py script performs a radiometric and terrain correction (RTC) using the methods described in [53]. RTC is important because RCS values vary with incidence angle and terrain, and those dependencies should be removed to better attribute RCS values to land cover and surface processes [54,55]. RTC processing can greatly reduce terrain impacts on RCS values and makes it possible for radar data obtained from different observation geometries to be interoperable with one another [56]. The ISCE script uses Sentinel-1 GRD data obtained from ASF Vertex as input and uses Sentinel-1 orbit information to accurately project data back into radar coordinates. Data were multi-looked three times in azimuth and range, resulting in 30 m × 30 m pixels. RTC processing requires a digital elevation model (DEM) to correct the RCS values. The accuracy of the DEM and the choice of the terrain-flattening approach impact the quality of the RTC result [26,54]. We use the Copernicus global 30 m DEM (GLO-30) as input to the terrain flattening workflow. GLO-30 is a new and relatively accurate DEM product having global coverage [57,58]. The ISCE-2 workflow uses the Ulander projection angle approach for terrain flattening [55]. Unlike previous studies that used 150 m × 150 m pixels [43], we used 30 m × 30 m pixels to obtain finer CA results and match the CDL resolution. However, the 30 m results had speckle noise artifacts, which can lead to classification errors. Therefore, a 7 × 7 enhanced lee speckle filter was used to further reduce noise [59]. Data were then co-registered to the CDL pixels. The study used data from 20 March to 16 November for years 2017 to 2021 to include planting and harvest periods that mainly focus on cash crops (see Figure 3 and Section 2.3). Data prior to 2017 were not used as coverage was temporally sparse. The study site was covered by Sentinel-1A in ascending mode (~6 p.m. local time) and located in ASF Frame 125, relative orbit 4.

The lack of data quality flags with SAR imagery poses a general challenge for large-scale SAR data processing. While not occurring often, data may be contaminated by artifacts unrelated to Earth surface processes, such as the suspected RFI shown in Figure 3a. This necessitates careful data screening, especially as the NISAR CA approach is, by design, sensitive to changes in RCS over time [37,41,43]. For example, the image artifacts only showed up in the processed CV values for 2017. Because removing the first RFI image did not remove all the artifacts in the CV stack, a second data quality check was conducted to remove the second date, resulting in a clean CV result image for 2017. This showcases one way for how the CV calculation can be utilized for data quality screening.

### 2.5. Developing a Binary Crop Map from the CDL

**Figure 4:**
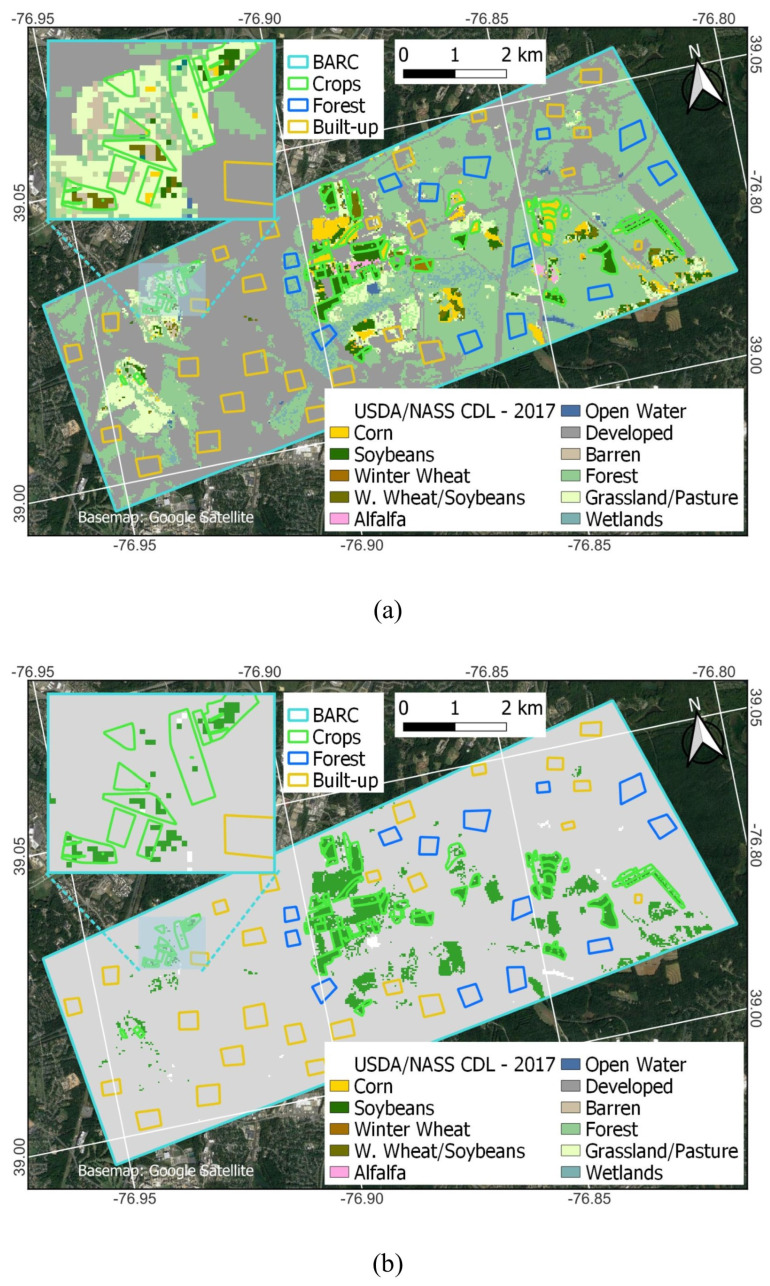
The 2017 CDL is colored by various CDL classes (**a**) and 2017 binary CDL with green, gray, and white showing crop, non-crop, and masked areas, respectively (**b**). (**a**) shows that the site consists of corn (yellow) and soybeans (dark green), according to the CDL.

The CDL includes many different crop and non-crop land cover classes. The CDL is produced once per year. The CDL runs classifications for all pixels first and then replaces non-crop classes using the 5-year NLCD. The CDL’s crop/non-crop map varies from year to year. For facilitating comparisons to CA, we translate the CDL into a binary crop/non-crop dataset according to [37]. All crops except tree crops are considered crop. Categories such as open water and aquaculture are masked because the coefficient of variation (CV) values (Equation (1)) are high and noisy over water, often showing as crop; in the case of clouds, it is because the CA does not have a land cover class value at those pixels for comparison against the CA result [43]. Tree crops are also masked because radar data is not expected to be capable of detecting comparable levels of change in RCS values compared to field crops and would consistently show as non-crop, as indicated by other studies [43,60]. The remainder is considered non-crop (e.g., developed, forest, wetland, and pasture/grassland), although CA has been shown to have some difficulty at consistently categorizing pasture, grassland, and developed land-use/land-cover areas as non-crop [43]. Figure 4 shows the CDL and binary CDL map for 2017. The CDL identifies roughly 5.5k out of 62k pixels (9%) categorized as cropland, depending on the year, similar to what is shown for 2017 in Figure 4b. According to the CDL, the most dominant non-crop land cover at BARC is built-up 27.4k (44%) pixels (gray in Figure 4a) and forest 22.4k (36%) pixels (green in Figure 4a). The remaining 6.7k (11%) pixels are other non-crop landcover types.

### 2.6. NISAR CA Approach

The NISAR CA approach used in this manuscript is the same as reported in prior work on this topic [37,39,43]. Data are co-registered and stacked in a time dimension on an annual cadence, ranging from 16 to 20 images per stack depending on year (Figure 3). Then, the coefficient of variation (CV) across time is calculated at each pixel (Equation (1)):

(1) $$CV=\frac{\sigma }{\mu }$$

where σ and µ are the temporal standard deviation and temporal mean of the data. Crop/non-crop classification is then determined using a threshold value for the CV (CV_thr_) at each pixel (CV_pixel_) as given in Equation (2):

(2) $${CV}_{pixel}\left\{\begin{array}{c} <{CV}_{thr}=0\\ \geq {CV}_{thr}=1 \end{array}\right.$$

with the assigned values of 0 and 1 corresponding to non-crop and crop, respectively. As mentioned in Section 2.2, the key premise of this approach is that agricultural land management (e.g., tilling, growth, and harvesting) would exhibit relatively greater change in RCS values over time compared to ‘constant’ areas such as forests [41]. Thus, crops usually have a high CV_pixel_ value, and a smaller CV_thr_ value increases crop area.

### 2.7. Threshold Selection

Receiver operating curve (ROC) approaches were used for identifying the optimal CV_thr_ value. Our work uses a different ‘look up’ approach, described in the last paragraph of this section. For completeness, the following describes the ROC-based approaches as well. The ROC requires a computation of the confusion matrix at small CV_thr_ increments. In prior work, 0.01 increments ranging from 0.0 to 1.0 had been used. It should be noted that CV values often exceed 1.00, but all prior studies so far indicated that the ideal CV_thr_ usually falls within a range of 0.2 to 0.7 for both L- and C- bands [38,39,43]. The confusion matrix is the result of the comparisons between the model (here, CA) to the ground truth (here, BARC FarmLogic) and consists of true positive (TP), false positive (FP), false negative (FN), and true negative (TN) detections.

The confusion matrix elements are then used to calculate Sensitivity (TP/(TP + FN)) and Specificity (TN/(TN + FP)) for each increment. To create the curve, data are then plotted using Sensitivity and 1-Specificity for the *y*-axis and *x*-axis, respectively. Earlier work also used histograms for identifying an optimal CV_thr_ value and compared it to ROC, showing nearly identical results [39]. More recent work, such as [37,38,43], also computed the kappa and the Youden J statistic at each CV_thr_ step and identified the CV_thr_ value corresponding to the maximum J statistic as optimal [61,62]. Iterative optimization approaches can be of value for local studies but are inefficient due to their repetitive nature, especially when attempting to map many regions, such as in Rose et al. (2021) [43].

**Figure 5:**
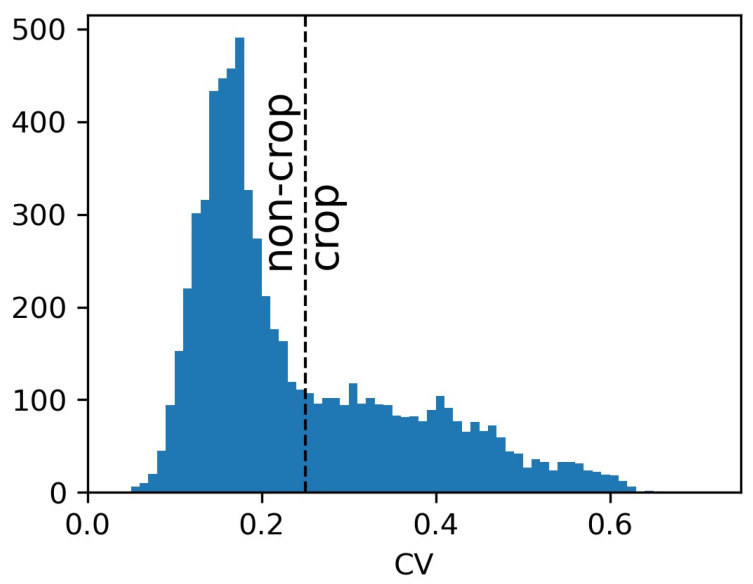
Histogram of 2017 CV values for crop, built-up, and forest fields. A vertical line is inserted at CV_thr_ = 0.25 to visually indicate its ability to distinguish between crop and non-crop classes. The bin size is 0.01.

Here, we use a different means of identifying a suitable CV_thr_ value, using information from relevant published studies. Rose et al. (2021) calculated optimal CV_thr_ values (maximum J statistic) over 100 1-by-1-degree tiles over CONUS, finding that optimal CV_thr_ varied in a somewhat gradual pattern across the United States, ranging from about 0.3 near coasts to about 0.5 in the center [43]. While that study only calculated results for 2017, other work reported that CV_thr_ values do not substantially vary in time [63], suggesting that 2017 CV_thr_ values would also be applicable to future years. Other previous studies plotted performance metrics (J statistic, kappa, and accuracy) as a function of CV_thr_, showing that performance metrics remained near optimal (~<5%) over a fairly wide CV_thr_ range, about ±0.1 from either side of the peak [37,42]. This means that it is sufficient to approximate a CV_thr_ corresponding to a high J statistic; an exact optimal value is not needed. Furthermore, [37] also showed that optimal CV_thr_ values tend to increase with finer grid spacing. That finding can be useful for translating the optimal CV_thr_ values across studies where they employ different spatial resolutions. Altogether, these prior findings present a means of identifying near-optimal CV_thr_ values a priori. First, we look up the value for a nearby location—i.e., the nearby sites reported by Rose et al. (2021) are located in North Carolina and Pennsylvania. Both had similar recommended CV_thr_ values in the 0.2 to 0.3 range [43]. Realizing that CV_thr_ values provided in [43] were obtained at a coarser resolution (150 m × 150 m) than this study uses (30 m × 30 m), according to [37] results, CV_thr_ values should be increased from 0.2 to reflect the finer pixels used here. This is also confirmed in Figure 5, the histogram of CV values for the fields used in this study. Figure 5 shows a distribution of two or more modes, with the major peak at CV = 0.17 (the non-crop fields). Selecting a CV_thr_ value of 0.20 would lead to more of the non-crop areas being misclassified as crop, supporting the idea of increasing the thresholds determined in the prior work using 150 m pixels.

### 2.8. Data Processing Framework and Assessment Methods

**Figure 6:**
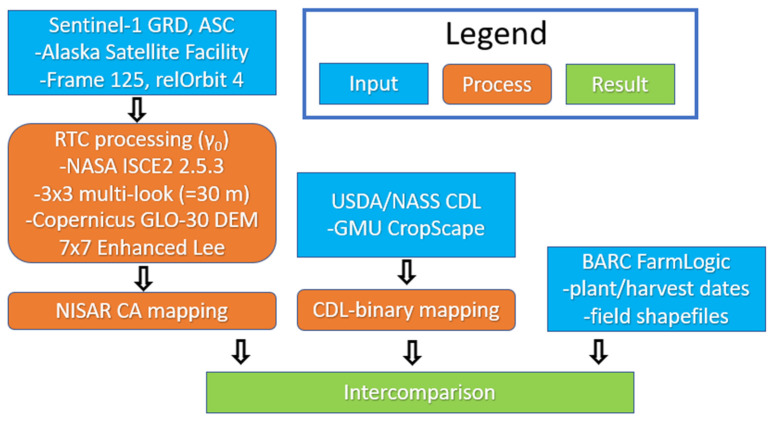
Data processing flow chart for NISAR CA and USDA/NASS CDL intercomparison with ground truth (BARC FarmLogic).

The processing and assessment methods used in this work are summarized in a flowchart (Figure 6). For this study, we produce the CA once per year using all the year’s available data (Figure 3) and applying a threshold of 0.25 (see Section 2.6 and Section 2.7). Areas that were masked in the CDL (Section 2.5) were also masked in the CA. The CA is a raster having three values—0 for non-crop, 1 for crop and a mask value used for areas outside the region of interest and the few masked land covers described in Section 2.5. As can be seen from the white areas in Figure 4b, barely any masking occurred: an average of only 128 CDL pixels inside the BARC boundary were masked per year out of about 62k BARC pixels.

**Figure 7:**
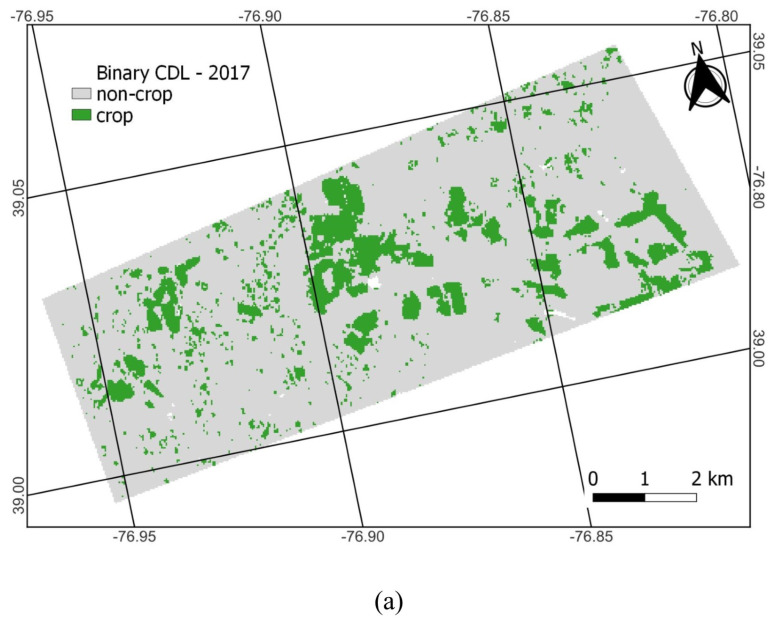

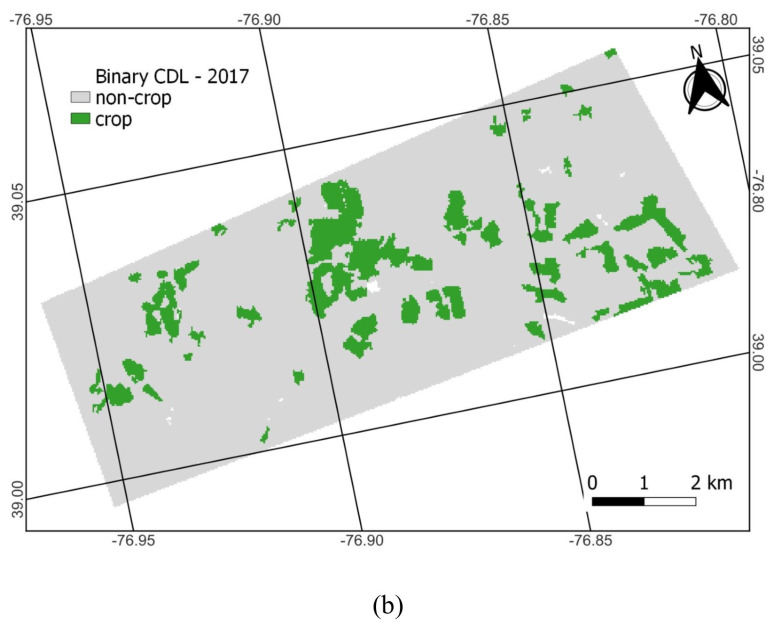
Result for the 2017 CA before (**a**) and after sieving (**b**), with white color indicating masked pixels.

We used a sieve operation implemented in the Geospatial Data Abstraction Software Library (GDAL) [64]. Sieving removes raster polygons smaller than the threshold size and replaces them with the pixel value of the largest neighbor polygon. We assessed sizes of none, 5, 10, 20, 50 and 100 pixels at four connectedness settling on a size of 20, which is shown in Figure 7b. Sieving is a common practice in removing noisy classifications and is also recommended for use with the CDL, although this study only applied the sieve to the CA [65].

The sieved CA was then intersected with the three types of polygons (crop, forest, built-up) shown in Figure 1. We only keep CA pixels (each having a value of 0 for non-crop or 1 for crop) where the pixel centroid fell inside the polygon field boundary. This result is useful for visually highlighting the degree of agreement for each boundary type, i.e., the degree to which polygons are correctly detected by CA as crop or non-crop. Next, the mean of all pixel values (0 for non-crop, 1 for crop) falling into each boundary is calculated. If the mean value was greater than 0.5, the boundary was classified as crop and was otherwise considered non-crop. We then compare the polygon label (ground truth) to the pixel-majority-based crop/non-crop classification result. A correct classification for crop, forest, and built-up boundaries was assessed to be crop, non-crop, and non-crop, and incorrect otherwise. Overall accuracy (OA) was then calculated as the number of correct classifications divided by total classifications (Table 2) [37]:

(3) $$OA=\frac{TP+TN}{TP+FP+FN+TN}*100$$

To investigate the degree of impact sieving had on results, we also repeated the assessments using the original CA map.

## 3. Results and Analysis

### 3.1. Pixel-Wise Correspondence between CA and CDL

Table 3 shows the annual OA when the CDL is used as ground truth for the CA using CV_thr_ = 0.25 for all pixels ahead of sieving and intersection with polygons. This is how most prior works were evaluated. OAs shown in Table 3 are within the range of values found in other studies using the CDL as ground truth in evaluations of the CA, showing that these two datasets have better than 85% agreement at this site every year.

### 3.2. Accuracy Assessment of CA and CDL versus Ground Truth Polygons

**Figure 8:**
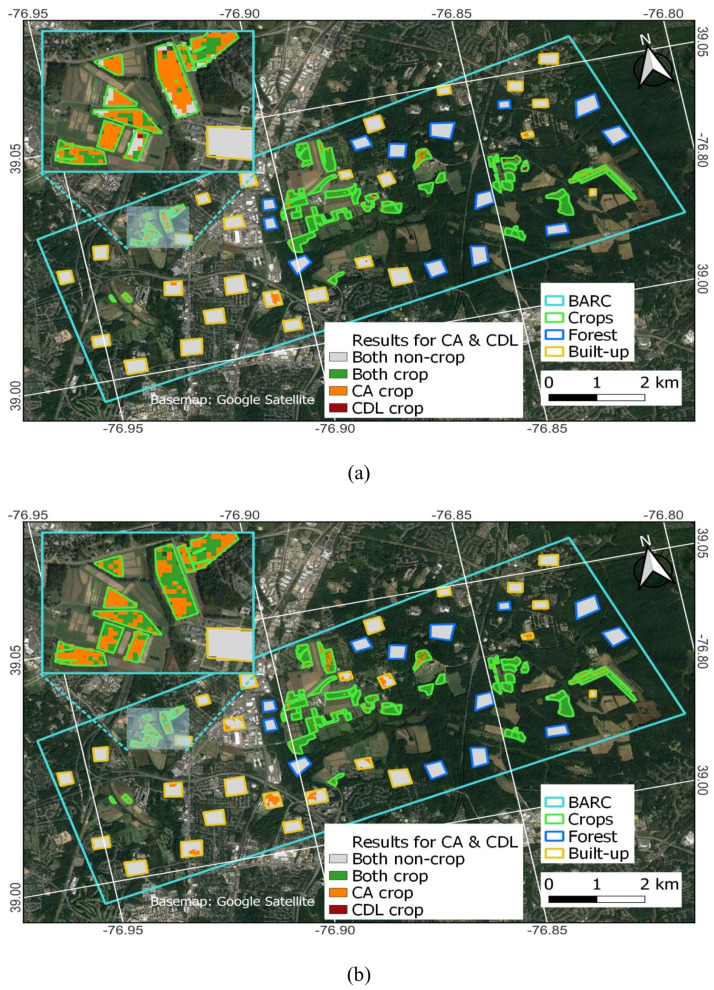

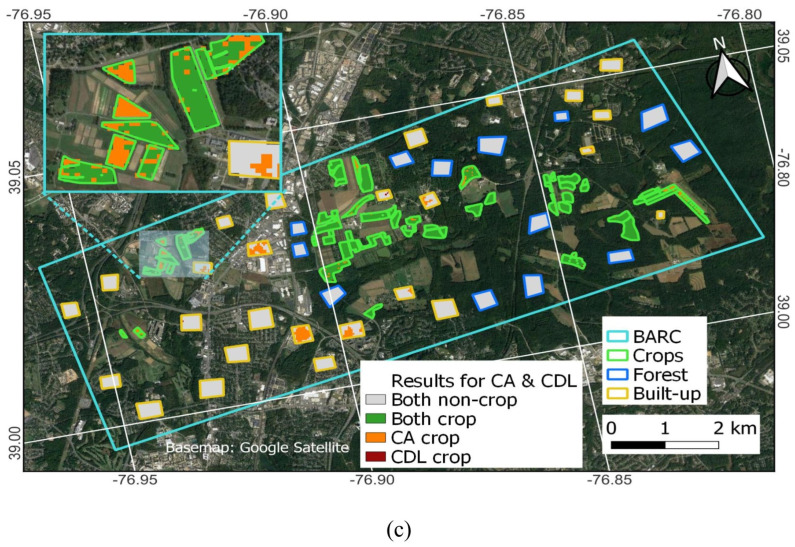
CA and CDL pixel-wise classification agreement for 2017 to 2019 (**a**–**c**) within the 93 polygons consisting of crop, built-up, and forest. Compared to the CA, the CDL has difficulty in correctly classifying pixels in the zoomed-in window.

**Figure 9:**
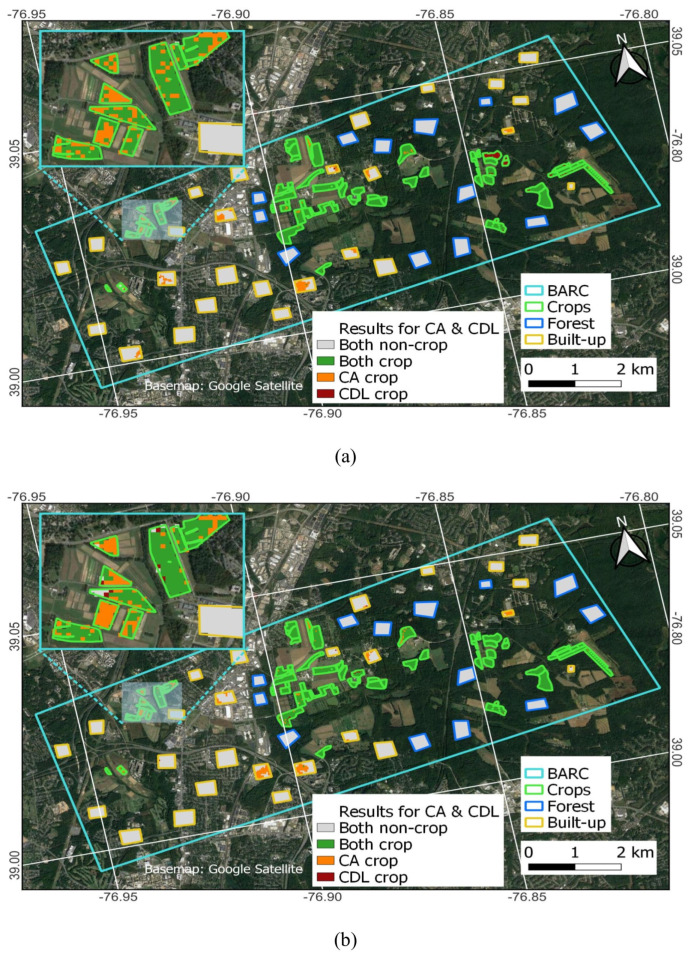
CA and CDL pixel-wise classification agreement for 2020 (**a**) and 2021 (**b**) within the 93 polygons consisting of crop, built-up and forest. Compared to the CA, the CDL has difficulty in correctly classifying pixels in the zoomed-in window.

Figure 8 and Figure 9 show the pixel-wise correspondence between CA and the CDL. Polygon edges are colored to provide a visual reference as to the ground truth status of the location: crop is green, built-up is orange, and forest is gray. While Table 3 indicates high correspondence between CDL and CA, Figure 8 and Figure 9 more clearly show the strengths and weaknesses of either approach. The strengths of the CDL lie in accurately identifying built-up and forest as non-crop in all years. However, the CDL has some difficulty in correctly classifying crop fields as crop, especially in 2017 and 2018. For example, there are many orange-colored pixels inside green polygons, especially in western fields for 2017 and 2018. While the CDL accuracy improves in 2019 and the following years, there remain several actively used fields that are not detected as crop in the CDL. The strengths of the CA lie in accurately identifying forest as non-crop, and crop as crop. However, the CA consistently detects a non-negligible amount of crop pixels inside some of the built-up polygons.

Consistent with the visual interpretation of Figure 8 and Figure 9, Table 4 shows that the CDL and CA both accurately categorize non-crop polygons. While the CDL achieves 100% accuracy for forest and built-up, the CA achieves 100% and 94% for forest and built-up, respectively. The greatest difference between the two datasets lies in the crop polygon classifications, where the CDL only averaged 76.7% compared to 95.6% for the CA. The CDL had considerable difficulty in accurately classifying many crop polygons in 2017 and 2018, only achieving 63%. This improved to over 85% in 2019 and later; however, the CDL was not able to exceed the CA crop detection performance in any year. It is unclear what specifically may have prompted the substantial improvement in CDL starting in 2019, but the USDA/NASS implemented some changes in creating the CDL at that time, such as applying smaller inward buffers to the CLU data ahead of rasterizing (personal communication, USDA/NASS 2023). The table also includes results for the CA before results were sieved (‘CA_ns_’) to explore how this operation impacted classifications. We find that the sieving did not appreciably impact OA (Table 4). The main reason for this was that although there were more misclassified pixels in CA_ns_, results did not exceed the 0.5 threshold needed for any of the polygons to change classification. Because sieving removes raster polygons smaller than the threshold size and replaces them with the pixel value of the largest neighbor cluster, sieving can increase crop/non-crop regions, depending on cluster location. The improvement in OA_crop_ for CA_ns_ corresponds to two more crop fields being detected. This is where sieving removed some crop pixels inside two crop polygons and fell below the 0.5 threshold, resulting in their classification as non-crop in CA (Central Farm 1-20C and South Farm SE 1-8-F). The improvement in OA_built-up_ corresponds to one additional built-up polygon being correctly classified as non-crop. Here, sieving filled in some non-crop pixels with crop pixels, resulting in the misclassification at the built-up polygon described as office park and restaurants in Section 4.3.

## 4. Discussion

### 4.1. Challenges for Cropland Mapping Using Spaceborne Radar Data

There are several challenges in cropland mapping using radar data. The foremost are (1) the general lack of freely available and routinely collected global radar datasets and (2) the computational burden in data processing costs that users must bear. Currently, the only widely distributed and free radar data source is Sentinel-1 at the C-band. Fortunately, there are other missions planned that will ameliorate these challenges, such as the deployment of additional Sentinel-1 satellites, NASA’s NISAR mission and ESA’s Copernicus Radar Observation Systems for Europe in L-band (ROSE-L) mission near the end of the decade [16,19,66]. The latter two have the additional benefit of the data providers taking on some processing, providing users with imagery in ARD format.

However, a remaining potential omission is that it is unclear whether future radar data will have a quality flag for each pixel, similar to how optical ARDs are provided. Temporary atmospheric or human impacts can unduly impact the RCS detected at the sensor, such as heavy precipitation, RFI, and ionosphere effects and may need to be flagged. Incorporating error flags would ultimately be necessary for big-data cloud processing, as it is not practical for each user to create their own data quality screening approach or rely on manual data quality inspections of every result tile in big-data processing, such as was done in [32]. Without quality flags, analyses are bound to include poor data, and this could make it difficult to attribute the RCS values to surface processes. This shortcoming also impacted this study because two dates were not used in the computation, although many of the pixels in the imagery appeared to have good data quality. Further, for this analysis, we needed to assume all data not showing obvious artifacts were of good quality.

### 4.2. Challenges for CDL Mapping

The CDL is a useful dataset because it provides great detail on crop type at desirable spatiotemporal resolutions. However, with so many different available crop classes, challenges in making correct crop-type (or even crop/non-crop) determinations are to be expected. The CDL’s main purpose is to inform on crop-type classifications rather than non-crop because the non-crop categories are imported from other data sources that may not be as frequently updated as the CDL. For this study, the misclassification of actively used agricultural fields as non-crop is a notable shortcoming. Looking in greater detail, the CDL misclassifications pre-2019 stem from the corn/soy fields in the western portion of the BARC campus being classified as grassland/pasture (the light green areas in Figure 4a), which becomes reclassified as a non-crop in the binary CDL (Section 2.5). Improvements from 2019 onwards in the CDL were mainly that the central field pixels were now correctly identified as crop (corn and soybeans).

The CDL data itself also has misclassification speckle, but unlike the CA, these tend to be confined to the field polygons. The Figure 4b inset map shows that crop areas have non-crop classes within or around their edges, indicating that cropland tends to be underestimated by the CDL at this location. Specifically, the inset shows there are six fields where there are a few correct crop classifications, but these generally tend to be limited to the center sections of the polygons. Most of the field edges are categorized as non-crop. Based on our results, there was no obvious need to implement additional preprocessing steps for the CDL, such as the sieve used for the CA. The CDL has two types of misclassification speckles: (1) pixels that are different crops and (2) pixels that are non-crop. In case (1), sieving would not impact the result because they are all crops. In case (2), sieving might still not help: it would depend on the pixel connectivity as to which pixels are reclassified, and the re-classifications would still need to result in crossing the 50% threshold to produce a different result for our methodology. Looking in more detail at Figure 4, 8 and 9 zoomed insets, even a priori, it is doubtful that sieving would change results too much for either the CDL or CA. For the CDL, looking at the bottom left field of Figure 4b inset shows that this would be correctly classified as crop (>50% crop pixels)—but crop pixels are split into two disconnected patches. Due to their small size, the sieve operation would remove both patches, changing the originally correct classification to an incorrect one when sieving. 

For the CDL, looking at the top right field in Figure 4b inset shows that it would be incorrectly classified as non-crop (<50% crop pixels). Given the pixel arrangement, sieving would not increase the crop pixel count in this polygon. This crop field would remain incorrectly classified as non-crop. This limited example shows that sieving the CDL may also negatively impact its OA, similar to what happened for the CA (CA_ns_ performed slightly better). It is important to emphasize that the overriding reason for recommending additional CA data processing steps (here, sieving) was primarily to produce more realistic non-crop mapping over built-up (Figure 7a vs. Figure 7b), rather than optimizing the reported OAs of this study. While the CDL data at this study site did not reveal a comparable need for implementing additional processing steps, it should be noted that some literature also recommends pre-processing the CDL prior to use [65].

Field size can be important for misclassifications, as fields may be too small in extent to be correctly detected as crop, especially when they have aspect ratios much different from unity. While FarmLogic data were screened by area, no consideration was given to aspect ratio. Figure 4, Figure 8, and Figure 9 show that some crop polygons can be narrow in one dimension, but each field is usually wide enough to contain at least two pixels in either dimension. Even so, this can make it challenging to correctly categorize the polygons, because each pixel would be considered a border pixel and could be mixed with an adjacent class. Thus, it can also be useful to consider the impacts of the polygon aspect on classifications in more detail. However, this is likely more a consideration for the use of CDL rather than CA, given that the study revealed that nearly all crop polygons were correctly detected by CA even when no screening for aspect was conducted.

Comparing the CA result in 2017 (Figure 7) and the CDL (Figure 4a) to BARC FarmLogic (Figure 1) shows that CA did not have substantial difficulty in detecting any of the crop areas even at >0.81 ha field size. This is consistent with estimates by [46] indicating that 30 m × 30 m satellite data should be sufficient at the scales of the polygons used in this study. The CDL uses some inward buffering of CLU data (30 m but only 15 m since 2019) to avoid mixed pixels ahead of rasterizing the CLU and selecting training and validation pixels. While this change in buffering temporally coincides with the improvement noticed starting in 2019, it is unclear how this alone would translate to the CDL’s improved results; there may have been other improvements starting in 2019.

### 4.3. Challenges for CA Mapping

The CA has good accuracy but with some noted difficulties for built-up polygons. This is consistent with CA results reported in a few other studies that also encompassed built-up pixels. Whelen and Siqueira (2017) applied the CA using L- and C-band data from the 2006 AgriSAR study (Germany), showing that histograms of CV values over urban areas have a wide-spread overlap with both crop (here, maize) and non-crop (forest) classes. They also noted scattered misclassifications over urban areas, suggesting that this may be due to the integration of small fields and gardens between buildings, which were categorized as non-crop in their reference dataset [41]. Kraatz et al. (2021) applied the CA to Sentinel-1 data over an agricultural site in Carman, Canada, and also showed that CV values for urban areas had considerable overlap with both crop and non-crop categories [38]. As a result, the CA performed poorly in correctly classifying built-up areas as non-crop in that study, only achieving slightly over 50% accuracy. Our study showed that CA could also have excellent performance over built-up areas, given that only about five out of twenty-six built-up polygons had a notable proportion of pixels misclassified as crop, with about two of them usually misclassified in any given year. This shows that CA results can vary considerably between locations.

**Figure 10:**
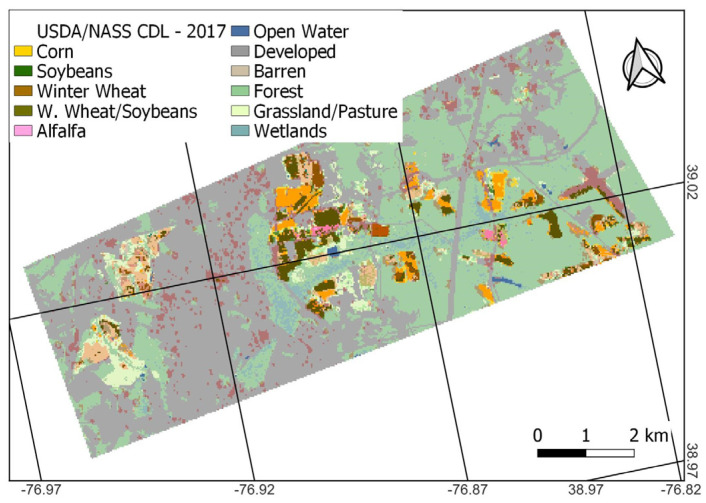
CA overlain onto the CDL for 2017. CA is recolored to show crop as semi-transparent red to facilitate the identification of speckle regions.

However, there are several key differences between the Carman and BARC studies. One difference is that the Carman study employed pixel-wise comparisons, while this study assesses accuracy according to correct polygon classifications. Another major difference is that the prevalence of non-crop pixels was smaller over the Carman study (~31%) compared to BARC (~91%). Specifically, Carman contained much fewer built-up pixels (5% of total) than this study (44%). Also, CV values were more dynamic over Carman, as evidenced in that study’s optimal CV_thr_ value being much larger (0.56) than here (0.25). Additionally, the Carman study used a different land cover reference dataset for evaluations, the Agriculture and Agri-Food Canada Annual Cropland Inventory [10]. Furthermore, the Carman study did not implement a sieve operation, which would have been able to remove the isolated higher CV locations (i.e., classification speckle) over the urban area. To elaborate further, Figure 10 clearly shows that the majority of CA’s misclassifications occurred over the built-up areas, but the sieve filter was able to remove most of them (Figure 7). Comparing results to Google satellite imagery, we determined that the larger speckle remaining after sieving corresponds to paved areas, including parking lots, office parks, shopping areas and restaurants. We also note that one of these larger patches (at −76.92, 39.02) is an agricultural field (Linkage Farm EB-3) that was not used in this study because it did not meet the screening criteria described in Section 2.3.

Overall, the CA classified about 17% of all pixels as crop compared to the CDL’s 9%, which helps explain the CA’s better ability to detect crop. This difference in crop pixel detections stems from two sources: overestimates of the crop by the CA at built-up areas and underestimates of crop by the CDL—i.e., the substantial number of non-crop pixels in or around the fields, but also any valid agricultural field pixels missed by the CDL but captured in the CA (Section 4.2).

**Figure 11:**
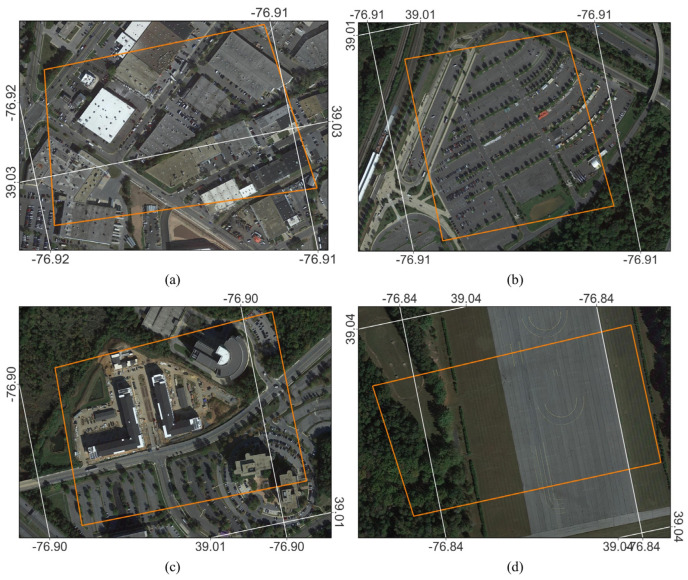
Zoom-in of the four built-up areas that were most frequently misclassified by CA as crop. These sites can also be viewed in the zoomed-out maps in Figure 1, Figure 4, Figure 8, and Figure 9. Areas containing auto repair and home improvement stores (**a**), Greenbelt Metro parking (**b**), office parks and restaurants (**c**), and part of a small airstrip (**d**).

We noted that a select few built-up polygons incorporate consistently misclassified pixels as crop for most years. This suggests that there may be some specific features of these locations that consistently give rise to misclassified pixels. We noted that two of the four sites include buildings and that all of the sites are paved (Figure 11). Figure 11 sites consist of office parks, restaurants, stores, metro parking, and an airstrip. Buildings are well known to cause a double bounce effect; strong radar returns occur where the signal reflects from a flat surface toward a building and back toward the detector. This is influenced by building heights and orientations relative to the satellite viewing geometry [67,68]. Sentinel-1 orbits differ over time, and baselines for the same frame and orbit may vary by up to about 200 m [32]. These variations in sensor–target geometry may result in variations in the strength of the double bounce effect for a given pixel containing buildings and, thus, higher CV values.

**Figure 12:**
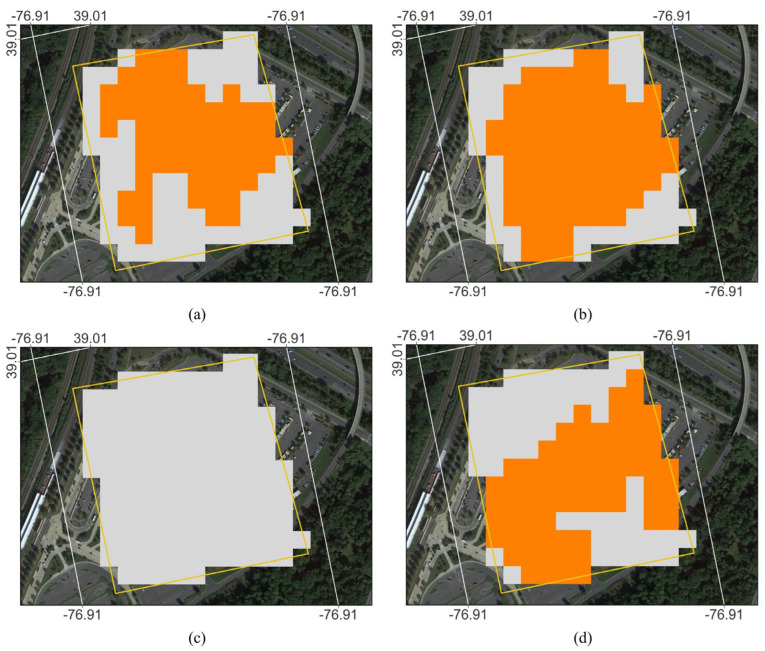
Zoom-in for the classifications over the Greenbelt Metro parking lot shown in Figure 11b, with the classification results for 2018 (**a**), 2019 (**b**), 2020 (**c**) and 2021 (**d**). The gray pixels show where the CDL and CA both correctly detected this non-crop area as non-crop. The orange pixels show where the CDL correctly detected non-crop, but the CA incorrectly detected crop.

Vehicles are also likely to be present at the Figure 11 sites when Sentinel-1 collects data at 6 p.m. It is possible that changes in vehicle presence also impact RCS sufficiently to produce false crop detections. This idea would be consistent with prior work that studied Sentinel-1 data over parking lots and with our relatively poorer results over the metro parking lot site shown in Figure 11b (see Section 3.2) [69]. We note that this study period also includes the onset of the COVID pandemic (early 2020) when the most severe travel, gathering and facility requirements were implemented. Of the four highlighted sites in Figure 11, the Metro parking (Figure 11d) is most likely to hold information on parking lot impacts on CA mapping in general and also whether the impact of COVID travel restrictions could be shown in this CA dataset. Coinciding with the pandemic onset and most severe travel restrictions, 2020 was the only year where all pixels in the Greenbelt Metro station polygon (Figure 11b) were correctly classified by CA (Figure 12). This observation can be cross-checked with data from the Washington Metropolitan Area Transit Authority (WMATA). WMATA publicly reports details on ridership and parking transactions by the station as average daily values for any given month, showing a large decrease in average daily parking transactions after March 2020 [70]. Averaging for the CA period (April–October), parking transactions decreased from 1686 in 2019 to 54 in 2020. Parking transactions have slowly been inching back toward pre-pandemic levels (110 in 2021 and 540 in 2022) ever since. While these data are, in principle, consistent with the idea of parking lot activity impacting CA results, we also note that the polygon was poorly classified by CA in 2021, even though 2021 exhibited a relatively slight uptick in daily transactions in absolute terms (56). Unfortunately, the scope of this study is not well-suited to study this potential relationship in further detail and make any more confident determinations. However, owing to the backdrop of wide availability of Sentinel-1 data, severe travel restrictions during COVID, plentiful large parking lots in CONUS and the CA’s potential sensitivity to human activity in them, considerably more detailed studies on this could be conducted in the future.

Although OA_ns_ performed slightly better, we recommend sieving CA data ahead of use when built-up areas constitute a substantial proportion of the study area. Using more numerous, smaller polygons for built-up areas and forests may give more detail in terms of how OA results are impacted by sieving. However, this was not revisited in greater detail as the polygon sizes used here are well within the normal range of agricultural fields in the region (Section 2.3), and sufficient information on the erroneous pixels was already provided between Section 2.8, Section 3.2 and Section 4.3 (Figure 7, Figure 8, Figure 9 and Figure 10).

### 4.4. Extension of the CA Algorithm to Other Regions

A major challenge for the CA lies in the selection of the CV_thr_ value used for delineating crop and non-crop. Smaller CV_thr_ values increase crop area, but also increase misclassifications over non-crop regions, whereas greater CV_thr_ values decrease crop area and increase misclassifications of agricultural fields. While prior work found that using a single threshold of CV_thr_ = 0.5 yielded >80% OA when applied to many different agricultural regions in CONUS [39,43], we used a considerably smaller CV_thr_ of 0.25. This is consistent with the results of [43], where a histogram of optimal CV_thr_ values over CONUS showed a bimodal distribution with peaks at about CV_thr_ = 0.3 and CV_thr_ = 0.5. Using the general recommendation of CV_thr_ = 0.5 would not be a useful choice for this study site, as CV values over BARC are much lower than other parts of CONUS, and many agricultural fields would not be detected. Elaborating further, given that only 9% of the pixels in the study area are crops, it is easy to obtain a 91% OA when selecting unreasonably large CV_thr_ values for this site (e.g., 0.5 or 1.0), resulting in all pixels being classified as non-crop. The intent of any cropland mapping study should be to put forth the best effort in the accurate detection of both crops and non-crops. Although only so much can be achieved using a single cutoff threshold, this will be improved when using CV_thr_ values corresponding to other performance metrics such as kappa and the J statistic peak. Rose et al. (2021) reported that the J statistic peaked between 0.2 and 0.3 at nearby locations (Section 2.7), and the results of this work confirmed that this threshold value provided an accurate classification of both crop and non-crop areas [43]. However, for routine and large-scale CA mapping, it is not feasible to determine the threshold for each study area and temporal subset to be used (e.g., annual, quarterly, bimonthly, etc.). Drawing from synergistic information from prior work, this study determined CV_thr_ values a priori (Section 2.7), which may be valuable for generalizing the CA over large spatial scales.

## 5. Conclusions

This work compared optical and radar-based cropland area mapping approaches against an extensive ground truth dataset (FarmLogic) over the Beltsville Agricultural Research Center (BARC) located in Beltsville, Maryland, USA. The ground truth data consisted of planting and harvesting dates, methods, and polygons of the agricultural fields. The BARC site also encompasses many built-up and forested areas, and separate polygons for these landcover types were generated to assist in the accuracy evaluations. The USDA/NASS Cropland Data Layer (CDL), an optical-based dataset, was re-classified into crop and non-crop for comparisons. The raster data were intersected with the reference polygons, with the pixel classification majority determining whether a polygon was crop or non-crop. The CDL achieved 100% classification accuracy over built-up and forest polygons. Accuracy over crop polygons was only 76.7%, resulting in an 86.5% overall accuracy. We note that while the CDL only detected 63% of fields in 2017 and 2018, this improved to over 85% for 2019–2021. The radar-based cropland area mapping approach used in this study (CA) is the same as that for the upcoming NISAR mission (operating at L-band and S-band), except that Sentinel-1 C-band data were used. Here, we provide a rule of thumb for looking up a crop/non-crop delineating threshold. There was no need for any training or calibration using this approach. CA classifications resulted in speckle noise over built-up areas, and a sieve filter was used. The CA achieved an overall accuracy of 95.7% and was 93.8%, 100%, and 95.6% accurate at identifying the built-up, forest, and crop polygons, respectively. Although the accuracy of the built-up polygons was high, several specific polygons regularly contained many misclassified pixels. The challenge of using the CA over built-up areas has been reported on in prior work, where it was suggested that errors stem from smaller fields or gardens. However, our most problematic built-up locations were those containing office parks, stores, restaurants, and parking lots. Results show that radar-based cropland (crop/non-crop) mapping is competitive with optical approaches, with added advantages in that it does not require training data, is operable under cloudy conditions, and has a lower computational cost.

## Figures and Tables

**Table 1:** Datasets used in this study.

Dataset/Tool	Institution	Avail.	Link
Farm Operations/shapefiles	USDA-BARC	open	https://doi.org/10.5281/zenodo.8087301 (accessed on 1 August 2023)
Sentinel-1 radar data	ESA	open	https://asf.alaska.edu/ (accessed on 1 August 2023)
Cropland Data Layer	USDA/NASS	open	https://nassgeodata.gmu.edu (accessed on 1 August 2023)
InSAR Computing Env.	NASA	open	https://github.com/isce-framework/isce2 (accessed on 1 August 2023)
Copernicus DEM	ESA	open	https://registry.opendata.aws/copernicus-dem/ (accessed on 1 August 2023)
WMTA ridership	WMTA	open	www.wmata.com (accessed on 1 August 2023)

**Table 2:** Confusion Matrix.

	Reference (BARC FarmLogic)	
Model (CA)	Crop	Non-crop
Crop	TP	FP
Non-crop	FN	TN

**Table 3:** Overall Accuracy (OA) between CDL and CA by year for CV_thr_ = 0.25. Results are pixel-wise and ahead of sieving and intersection with polygons.

Year	OA (%)
2017	88.7
2018	85.3
2019	86.4
2020	87.6
2021	87.6
AVG	87.1

**Table 4:** Accuracy of CDL and CA against ground truth (BARC FarmLogic) by polygon category and overall. Also included is the tally for CA without sieving (CA_ns_).

Year	OA_crop,n=54_ (%)			OA_built-up,n=26_ (%)			OA_forest,n=13_ (%)		OA_all,n=93_ (%)		
	CDL	CA	CA_ns_	CDL	CA	CA_ns_	CDL	CA, CA_ns_ *	CDL	CA	CA_ns_
2017	63.0	88.9	92.6	100.0	100.0	100.0	100.0	100.0	78.5	93.5	95.7
2018	63.0	100.0	100.0	100.0	92.3	92.3	100.0	100.0	78.5	97.8	97.8
2019	85.2	100.0	100.0	100.0	92.3	92.3	100.0	100.0	91.4	97.8	97.8
2020	85.2	88.9	88.9	100.0	92.3	96.2	100.0	100.0	91.4	91.4	92.5
2021	87.0	100.0	100.0	100.0	92.3	92.3	100.0	100.0	92.5	97.8	97.8
AVG	76.7	95.6	96.3	100.0	93.8	94.6	100.0	100.0	86.5	95.7	96.3

* The CA and CA_ns_ results are combined because they are identical.

## Data Availability

Data and tools supporting the conclusions and data processing for this manuscript are available online. Sentinel-1 Ground Range Detected data used for input to the NISAR cropland mapping approach were downloaded from the Alaska Satellite Facility (ASF) (https://search.asf.alaska.edu, accessed on 1 August 2023) with the access condition of using an Earthdata account (https://urs.earthdata.nasa.gov/users/new, accessed on 1 August 2023). The dataset can be found by providing a polygon covering the study area coordinates, as shown in Section 2.2. The InSAR Scientific Computing Environment software (ISCE) version 2.5.3 was used to process the Sentinel-1 GRD data using the rtcApp.py script (https://github.com/isce-framework/isce2, accessed on 1 August 2023). An example workflow for the NISAR cropland mapping approach is available at https://github.com/UMassMIRSL/Coefficient_of_Variation_CropClassification (accessed on 1 August 2023). The BARC field data, including field shapefiles, region of interest and inventory, are available at Zenodo https://doi.org/10.5281/zenodo.8087301 (accessed on 1 August 2023).
